# Bilateral Optic Disc Edema in the Course of Idiopathic Intracranial Hypertension: A Case Report

**DOI:** 10.7759/cureus.87845

**Published:** 2025-07-13

**Authors:** Aleksandra Górska, Krzysztof Kuzniar, Malgorzata Kuzniar, Arlena Wyrebak

**Affiliations:** 1 Department of Ophthalmology, Professor K. Gibiński University Clinical Center, Medical University of Silesia in Katowice, Katowice, POL; 2 Department of Family Medicine, St. Michael Archangel Family Medicine Center Medicus, Katowice, POL; 3 Department of Otolaryngology and Subunit of Maxillofacial Surgery for Children, Municipal Hospital Complex, Chorzów, Katowice, POL; 4 Department of Anesthesiology, Professor K. Gibiński University Clinical Center, Medical University of Silesia in Katowice, Katowice, POL

**Keywords:** idiopathic intracranial hypertension, ihh, obesity, obesity-related illnesses, papilledema

## Abstract

Idiopathic intracranial hypertension (IIH) is a rare disease characterized by elevated cerebrospinal fluid pressure in the absence of structural changes in the brain. One of the main symptoms of IIH is deterioration of visual acuity with evidence of swelling of the optic nerve disc. Other complaints reported by patients may also include headaches, dizziness, and tinnitus.

A 46-year-old female patient presented to the ophthalmology outpatient clinic because of bilateral visual acuity deterioration. The patient had previously been treated for hypertension and dizziness, and she also reported increasing obesity over the past three months. A fundus examination revealed bilateral swelling of the optic disc with obliterated borders and elevation above the fundus level. An optical coherence tomography study was also performed. The patient was consulted neurologically. A CT scan and an MRI of the head were performed, showing no abnormalities. The collected cerebrospinal fluid showed an elevated pressure value. Based on the clinical picture, IIH was diagnosed. Acetazolamide (2 x 250 mg) was included in the treatment, with an improvement in visual acuity after one month of treatment. In addition, the patient was referred to the metabolic disease clinic for weight reduction.

The presented case is intended to highlight the need to consider IIH in patients with bilateral visual deterioration and optic disc edema. Early diagnosis and implementation of pharmacological treatment, as well as lifestyle modification with weight reduction, can prevent permanent complications in the form of permanent optic nerve damage, among others.

## Introduction

Idiopathic intracranial hypertension (IIH), also known as pseudotumor cerebri, is a rare condition characterized by elevated cerebrospinal fluid pressure without an obvious cause such as a brain tumor or venous sinus thrombosis. Some of the main symptoms of IIH are bilateral swelling of the optic nerve disc, which can lead to deterioration of visual acuity and in extreme cases, even loss of vision, transient visual impairment, which can be triggered by changes in body position, and double vision (diplopia), which is usually horizontal and caused by paralysis of the VIth cranial nerve. Other symptoms reported by patients may include headaches that worsen when lying down, dizziness, and tinnitus, which is usually unilateral and can be auscultated as a murmur synchronous with the heart rate.

In this article, we present a case of a 46-year-old female patient with bilateral optic disc edema in the course of IIH.

## Case presentation

**Figure 1:**
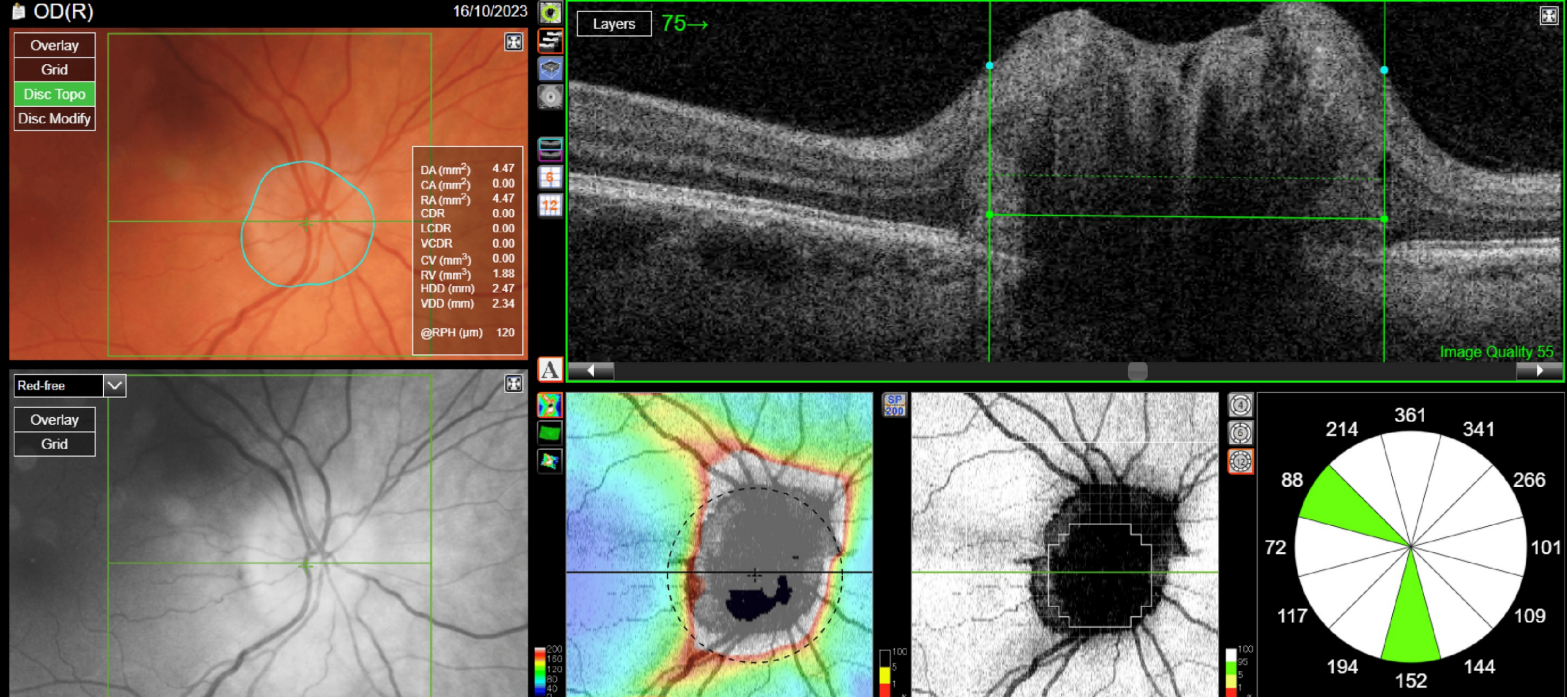
Optical coherence tomography of the optic nerve disc of the right eye before treatment.

**Figure 2:**
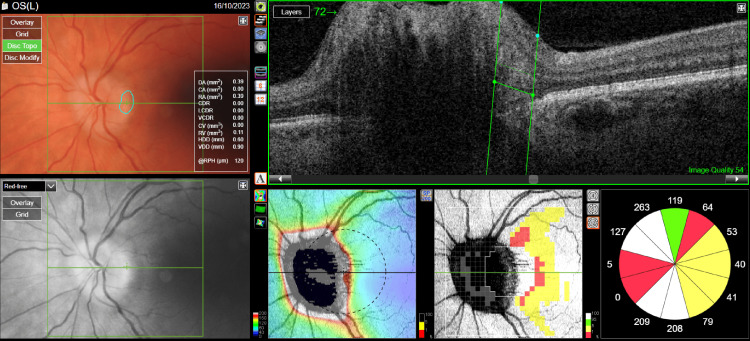
Optical coherence tomography of the optic nerve disc of the left eye before treatment.

A 46-year-old female patient presented to an ophthalmology outpatient clinic because of a bilateral deterioration of visual acuity that had persisted for about six months. She had a history of hypertension, obesity (rapid weight gain of 30 kg in three months), and dizziness for six years. A fundus examination showed bilateral swelling of the optic disc with obliterated borders and elevation above the fundus level (Figures 1, 2). Best-corrected visual acuity (BCVA) was 0.8 for the right eye and 0.7 for the left eye. The intraocular pressure of the right eye was 20 mmHg, and that of the left eye was 19 mmHg. Optical coherence tomography (OCT) was performed, and fundus photography was taken.

A computed tomography (CT) scan of the head without contrast and a magnetic resonance (MR) scan of the head with contrast were performed to expand the diagnosis, which showed no abnormalities. The patient was referred to the neurology department, where a lumbar puncture was performed. The cerebrospinal fluid (CSF) was watery, clear, with pleocytosis of 1, protein, and normal glycemia. The CSF pressure was 450 mmH₂O (normal for an obese person is up to 250 mmH₂O). Based on the findings, IIH was diagnosed.

**Figure 3:**
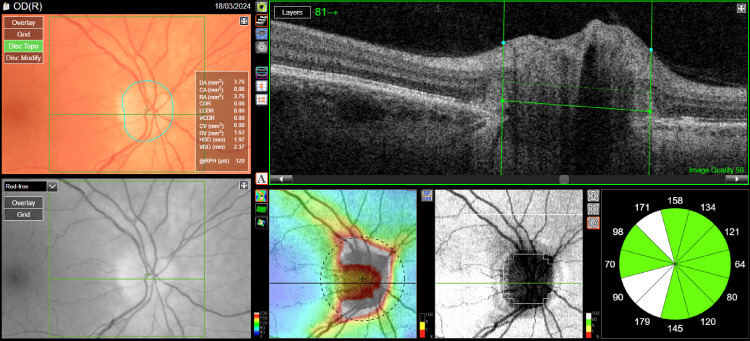
Optical coherence tomography of the optic nerve disc of the right eye after treatment.

**Figure 4:**
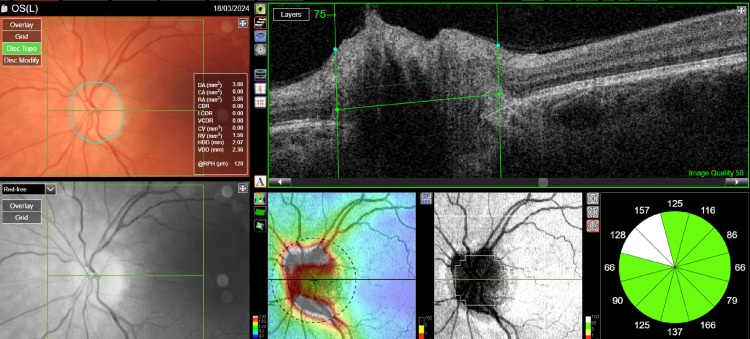
Optical coherence tomography of the optic nerve disc of the left eye after treatment.

Treatment with acetazolamide at a dose of 2 x 250 mg was started. After one month of treatment, visual acuity improved to 1.0 in both eyes, and disc edema decreased (Figures 3, 4). The patient remains under constant follow-up with the ophthalmology clinic, neurology clinic, and metabolic disease clinic.

## Discussion

IIH, also known as pseudotumor of the brain, is a rare but potentially dangerous condition that can lead to serious complications, including permanent optic nerve damage [1]. In the case of our 46-year-old patient, the key symptom that prompted further diagnosis was bilateral swelling of the optic disc. IIH occurs predominantly in young, obese women of childbearing age, as also confirmed by the case of our patient, who experienced a rapid weight gain of 30 kg in three months [2,3].

Imaging studies such as CT and MRI are crucial in the diagnosis of IIH to exclude other causes of elevated intracranial pressure, such as brain tumors, venous sinus thrombosis, or infections [4]. In our patient's case, both CT and MRI of the head showed no abnormalities, which confirmed the diagnosis of IIH. However, the most important test in the diagnosis of IIH is lumbar puncture with measurement of CSF pressure. In our patient, the CSF pressure was 450 mmH₂O (45 cmH₂O), which is markedly elevated compared to the normal range (50-150 mmH₂O in healthy individuals) and exceeds even the upper limit seen in obese patients with IIH (typically ≤250 mmH₂O) [5].

Treatment of IIH, depending on the severity of symptoms, is based on three main pillars: weight reduction, pharmacotherapy, and, in some cases, surgical intervention [6]. Acetazolamide, a carbonic anhydrase inhibitor, is the first-line treatment for moderately severe cases of IIH. It reduces cerebrospinal fluid production, leading to a reduction in intracranial pressure [7]. In our patient's case, treatment with acetazolamide was initiated, and after one month of therapy, a significant improvement in visual acuity from 0.7/0.8 to 1.0 was observed in both eyes. However, long-term treatment of IIH requires ongoing ophthalmic and neurological care to monitor disease progression and prevent recurrence [1].

The main complication of IIH is loss of vision due to swelling of the optic disc. This swelling leads to compression of ganglion cell axons, which can result in their death and permanent loss of visual function [8]. In our patient's case, the correct diagnosis and implemented treatment prevented these complications.

IIH is a condition with a complex etiology, and its pathophysiology is not fully understood. Abnormalities in CSF flow and increased resistance to CSF outflow are thought to play a key role [9]. In addition, hormonal factors, particularly in women, may play an important role in the development of IIH. In our patient's case, rapid weight gain may have been one of the triggers [10].

The literature highlights the importance of early diagnosis and treatment of IIH to prevent permanent optic nerve damage [1]. A study by Friedman et al. showed that early implementation of acetazolamide treatment significantly improves the prognosis of patients with IIH [4]. Similarly, Sinclair et al. highlighted that not only drug treatment but also weight reduction can lead to long-term remission of the disease [11].

IIH is a rare but serious condition that requires comprehensive diagnosis and treatment. In the case of our patient, early diagnosis and the implementation of acetazolamide treatment allowed a significant improvement in visual acuity and prevented further damage to the optic nerve. Imaging studies and measurement of cerebrospinal fluid pressure are crucial in the diagnosis of IIH, and treatment should include both pharmacotherapy and weight reduction [4,5].

## Conclusions

Idiopathic intracranial hypertension should be considered in the differential diagnosis of bilateral optic disc edema, especially in patients with obesity and other risk factors. Imaging studies and measurement of CSF pressure are crucial in making a correct diagnosis. Early implementation of treatment can prevent permanent optic nerve damage and improve patients' prognosis.
